# Obesity indices predict hypertension among indigenous adults in Krau Wildlife Reserve, Peninsular Malaysia

**DOI:** 10.1186/s41043-017-0102-4

**Published:** 2017-05-25

**Authors:** Ee Yin Chua, Mohd Shariff Zalilah, Karppaya Haemamalar, Sulaiman Norhasmah, Appannah Geeta

**Affiliations:** Department of Nutrition and Dietetics, Faculty of Medicine and Health Sciences, Universiti Putra Malaysia, 43400 UPM Serdang, Selangor Darul Ehsan Malaysia

**Keywords:** Obesity indices, Hypertension, Indigenous peoples, Waist-height ratio, Predictor

## Abstract

**Background:**

The disease burden of indigenous peoples has been augmented by the rising prevalence of obesity and hypertension in this population. This study assessed the ability of obesity indices to predict hypertension among indigenous adults of Peninsular Malaysia.

**Methods:**

In this cross-sectional study, 482 adults (223 men, 259 women) aged ≥18 years old were measured for body mass index (BMI), waist circumference (WC), waist-height ratio (WHtR), waist-hip ratio (WHR), and blood pressure. Receiver operating characteristic (ROC) analysis was used to determine the predictive ability of obesity indices for hypertension in men and women. Gender-specific logistic regression analyses were done to examine the association between obesity, defined by BMI, WC, WHtR and WHR, and hypertension.

**Results:**

Prevalence of hypertension was 25.5%. Overall, WHtR was the best predictor of the presence of hypertension, in both men and women. The optimal WHtR cut-off values for hypertension were 0.45 and 0.52 in men and women, respectively. Obese adults with WHtR ≥0.5 had about two times increased odds of having hypertension compared to non-obese adults.

**Conclusions:**

WHtR may serve as a simple and inexpensive screening tool to identify individuals with hypertension in this relatively difficult to reach population.

## Background

World Health Organization (WHO) reported that one in six adults is obese and one in three has elevated blood pressure [1]. Obesity and hypertension are among the preventable risk factors for cardiovascular diseases that impose a considerable economic burden, particularly in the developing countries [2]. Hypertension is ranked as one of the three leading risk factors for global disease burden, followed by tobacco smoking and alcohol use [3]. It is also reported to be more prevalent in low- and middle-income countries, and the number of people with undiagnosed, untreated, and uncontrolled hypertension are higher in these countries compared to high-income countries [4]. As obesity increases the risk of hypertension, addressing the obesity and hypertension epidemic is crucial.

Indigenous peoples experience both socioeconomic and health disadvantages [5–7]. Although indigenous peoples constitute less than 5% of the global population, they account for almost 15% of poor people worldwide [8]. Food systems of indigenous peoples are very much influenced by economic and ecological changes [9]. Historically, indigenous peoples depended on local natural resources obtained through hunting, fishing, and subsistence farming. In recent years, rapid economic growth has increased indigenous peoples’ employment opportunities and consequently dependence on market foods. However, indigenous peoples remain to be the poorest or most disadvantaged population. With limited income, they are more likely to have restricted food choices, particularly nutritious foods. Increased access to energy dense and nutrient-poor market food which are relatively cheap and the reduced consumption of traditional food is associated with increased risk of obesity and chronic diseases among the indigenous peoples [10, 11]. Lack of access to health care services, particularly for those residing in remote areas, further increases the risk of chronic diseases in this population [12].

In Malaysia, the National Health and Morbidity Survey (NHMS) showed an increasing trend in obesity, abdominal obesity, and hypertension among adults [13, 14]. Prevalences of obesity and abdominal obesity have increased from 14% (2006) to 15.1% (2011) and from 39.5% (2006) to 45.4% (2011), respectively. During the same period, the prevalence of hypertension has increased from 32.2% (2006) to 32.7% (2011). The prevalence of undiagnosed hypertension was found to be highest in indigenous peoples and higher in rural than in urban areas [14]. Hypertension and it complications are costly, and underdiagnosis of hypertension may exacerbate the disease burden of the population.

Orang Asli are non-Malay indigenous peoples of Peninsular Malaysia, and they constitute less than 1% of the total population [15]. Most of them are poor and live in rural or remote areas. Earlier studies reported that chronic energy deficiency was prevalent in the Orang Asli adult population [16–18]. Besides, stunting and underweight were common undernutrition problems among the Orang Asli children [19–21]. However, over the years, overweight and obesity have emerged as a health concern in the Orang Asli adult population. Cross-sectional studies showed the prevalence of adult overweight and obesity was about 20 to 50% [22–25]. While undernutrition persists among the Orang Asli, particularly children, there is a coexistence of overweight and obesity in the adults which could increase their risk of chronic diseases.

Anthropometric measures, including body mass index (BMI), waist circumference (WC), waist-hip ratio (WHR), and waist-height ratio (WHtR), are commonly used as a proxy measure of obesity. While BMI reflects overall obesity, WC, WHR, and WHtR indicate abdominal obesity. Given that overweight and obese people have a higher risk of hypertension and anthropometric measurements are relatively easy and inexpensive to perform [26–28], numerous attempts have been made to predict hypertension based on obesity indices [29–31]. However, there is a lack of consensus on the best predictive indicator of hypertension. As obesity and its associated non-communicable diseases become a health challenge in this difficult to reach population, there is a need to identify a simple measure of obesity that can be used to screen for individual at risk of hypertension. Therefore, this study aimed to determine and compare the ability of obesity indices (BMI, WC, WHtR, and WHR) to predict hypertension and to identify the best predictor of hypertension among the Orang Asli adult population.

## Methods

### Study subjects

This cross-sectional study was conducted in 20 Orang Asli villages in Krau Wildlife Reserve, Pahang, Peninsular Malaysia. Men and women aged 18 years old and above were included while pregnant and lactating women and those who were bedridden or with physical disabilities were excluded from this study. Based on their clinic/hospital cards, individuals with current or prior history of chronic diseases such as cancer, cardiovascular disease, diabetes, and renal diseases were excluded while individuals with known hypertension or hyperlipidemia and on medications but did not have the aforementioned chronic diseases were included in the study. Out of 376 households in the 20 villages, a total of 482 adults from 252 households (154 Jah Hut, 67 Temuan, and 31 Che Wong) consented to participate in the study.

### Measurements

Respondents were interviewed in the home setting using a pre-tested questionnaire for information on socio-demographic information, smoking habit, and alcohol consumption. Those who were currently smoking or consuming alcohol either regularly or occasionally (over the past 1 month) were defined as “smoker” and “drinker,” respectively. Anthropometry and blood pressure were measured by trained enumerators.

### Anthropometry

Height, weight, and hip and waist circumferences of respondents were measured using the SECA 213 portable stadiometer, TANITA HD 314 digital weighing scale, and SECA 201 measuring tape, respectively. Each measurement was taken twice, and the average value was used to calculate BMI, WHR, and WHtR. Obesity was defined as BMI ≥30 kg/m^2^ and WHtR ≥0.5 for both men and women, WC ≥90 cm and WHR ≥0.9 for men and WC ≥80 cm and WHR ≥0.85 for women [32–34].

### Blood pressure

Systolic blood pressure (SBP) and diastolic blood pressure (DBP) were measured using an Omron BP710 Automatic Blood Pressure Monitor after each respondent had rested for at least 10 min in a sitting position. Blood pressure was measured twice on the left arm, at 30-s intervals, with an appropriately sized cuff, and the average value was recorded. Individuals with SBP ≥140 mmHg and/or DBP ≥90 mmHg, or individuals on anti-hypertensive medication, based on their clinic or hospital cards, were defined as hypertensive [35].

### Statistical analysis

Statistical analyses were performed using MedCalc version 16.1 (MedCalc Software, Ostend, Belgium). Descriptive statistics such as mean and percentage were used to describe the data. Continuous variables were tested for normality. Comparisons of characteristics by sex were made using independent *t* test and chi-square test for normally distributed continuous variables and categorical variables, respectively. Sex-specific receiver operating characteristic (ROC) curves were plotted to examine and compare the abilities of obesity indices to predict hypertension. The area under the ROC curve (AUC) was used to determine the ability of a particular obesity index to predict hypertension. An AUC of 1 reflects a perfect predictive power, and an AUC of 0.5 indicates that the predictive power is no better than chance. The AUCs were compared to one another using the DeLong method [36]. An optimal cut-off point for ROC curve of corresponding obesity index was determined to maximize sensitivity and specificity and was estimated using the highest Youden index. Logistic regression was conducted to examine the association between hypertension and obesity status, defined by BMI, WC, WHtR, and WHR, for men and women separately. Age, smoking status, alcohol consumption, and ethnicity (sub-tribes) were treated as covariates in the adjusted models. Statistical significance was set at *p* < 0.05.

## Results

The sample comprised 223 men (46.3%) and 259 women (53.7%) with a mean age of 35.4 years (Table 1). About 87.3% of households were classified as either poor (household monthly income <RM870) or hardcore poor (household monthly income <RM580) [37]. More than one fifth (22.6%) of Orang Asli adults consumed alcohol whereas more than half (53.3%) smoked in the past 1 month. Approximately 30% of Orang Asli adults were either overweight or obese, and the proportion of obese women was four times higher than men. About 45.4% of the adults (34.1% of men; 55.2% of women) were centrally obese, classified as having either at risk WC or WHtR or even WHR. More men and women were categorized as centrally obese based on WHtR criterion as compared to WC and WHR. Meanwhile, 25.5% of adults were hypertensive with higher prevalence in men (31.4) than women (20.5). Out of this number, 20 (16.3%) were medically diagnosed but only 8 (6.5%) were on anti-hypertensive medication.

**Table 1:** Characteristics of study subjects

Characteristics	Total (*N* = 482)		Men (*n* = 223)		Women (*n* = 259)		*p* value†
	*n* (%)	Mean [95% CI]	*n* (%)	Mean [95% CI]	*n* (%)	Mean [95% CI]	
Sub-tribe							0.965
Jah Hut	297 (61.6)		136 (61.0)		161 (62.2)		
Temuan	132 (27.4)		62 (27.8)		70 (27.0)		
Che Wong	53 (11.0)		25 (11.2)		28 (10.8)		
Age (years)		35.4 [34.3, 36.5]		37.5 [35.8, 39.1]		33.6 [32.2, 35.1]	<0.01
18–29	181 (37.5)		72 (32.3)		109 (42.1)		
30–39	123 (25.5)		57 (25.6)		66 (25.5)		
40–49	104 (21.6)		49 (22.0)		55 (21.2)		
≥50	74 (15.4)		45 (20.1)		29 (11.2)		
Drinker	109 (22.6)		101 (45.3)		8 (3.1)		<0.001
Smoker	257 (53.3)		160 (71.7)		97 (37.5)		<0.001
Anthropometric measures							
Weight (kg)		55.6 [54.5, 56.7]		58.4 [57.1, 59.7]		53.2 [51.5, 54.9]	<0.001
Height (cm)^a^		154.0 [153.3, 154.6]		159.1 [158.4, 159.9]		149.5 [148.8, 150.3]	<0.001
Short stature	276 (57.3)		126 (56.5)		150 (57.9)		
Normal height	206 (42.7)		97 (43.5)		109 (42.1)		
Hip circumference (cm)		92.0 [91.0, 93.0]		90.9 [90.7, 92.2]		93.0 [91.5, 94.5]	<0.05
Obesity indices							
Body mass index (BMI) (kg/m^2^)^b^		23.41 [22.97, 23.83]		23.03 [22.57, 23.48]		23.73 [23.03, 24.43]	0.098
Underweight	49 (10.2)		12 (5.4)		37 (14.2)		
Normal	288 (59.8)		159 (71.3)		129 (49.8)		
Overweight	106 (22.0)		45 (20.2)		61 (23.6)		
Obese	39 (8.0)		7 (3.1)		32 (12.4)		
Waist circumference (WC) (cm)^c^		76.6 [75.6, 76.7]		76.4 [75.0, 77.7]		76.9 [75.3, 78.4]	0.612
Normal	374 (77.6)		201 (90.1)		173 (66.8)		
At risk	108 (22.4)		22 (9.9)		86 (33.2)		
Waist-height ratio (WHtR)^d^		0.50 [0.49, 0.51]		0.48 [0.47, 0.49]		0.51 [0.50, 0.52]	<0.001
Normal	286 (59.3)		152 (68.2)		134 (51.7)		
At risk	196 (40.7)		71 (31.8)		125 (48.3)		
Waist-hip ratio (WHR)^e^		0.83 [0.83, 0.84]		0.84 [0.83, 0.85]		0.82 [0.81, 0.84]	0.082
Normal	349 (72.4)		184 (82.5)		165 (63.7)		
At risk	133 (27.6)		39 (17.5)		94 (36.3)		
Clinical parameters							<0.001
Systolic blood pressure (mmHg)		121 [120, 123]		126 [124, 129]		117 [115, 119]	<0.001
Diastolic blood pressure (mmHg)		81 [80, 82]		84 [82, 85]		80 [78, 81]	
Hypertension	123 (25.5)		70 (31.4)		53 (20.5)		<0.01

†Comparison between men and women

^a^Short stature (men ≤160 cm; women ≤150 cm); normal height (men >160 cm; women >150 cm)

^b^Underweight (BMI <18.50 kg/m^2^); normal (BMI 18.50–24.99 kg/m^2^); overweight (BMI 25.00–29. 99 kg/m^2^); obese (BMI ≥30 kg/m^2^)

^c^Normal (men <90 cm; women: <80 cm); at risk (men ≥90 cm; women ≥80 cm)

^d^Normal (<0.50); at risk (≥0.50)

^e^Normal (men <0.9; women <0.85); at risk (men ≥0.9; women ≥0.85)

The ability of each obesity index to predict hypertension is indicated as the area under the ROC curves (AUC) (Table 2). The AUC for obesity indices ranged from 0.55 to 0.66 and 0.59 to 0.67, in men and women, respectively. Of the obesity indices, WHtR followed by both WC and WHR yielded the highest AUC in men. For women, AUC for WHR was the highest, followed by WC and WHtR. Having the largest AUC, WHtR and WHR seemed to be the best predictor of hypertension for men and women, respectively. The optimal WHtR cut-off points were 0.45 and 0.52 for men and women, respectively. Meanwhile, the optimal WHR cut-off points for each male and female subject were 0.81 and 0.82. The differences in AUC among WC, WHtR and WHR in both sexes were not significant indicating that these indices were comparable in their predictive ability. BMI was the least predictive of hypertension for men and women. The optimal cut-off point varied by sex and differed from the corresponding established cut-off points for obesity.

**Table 2:** Predictive ability of obesity indices for hypertension

	AUC [95% CI]	Optimal cut-off point^a^	Youden index	Sensitivity (%)	Specificity (%)
Men					
BMI	0.55 [0.47, 0.64]^b,c^	23.54	0.15	51.4	64.1
WC	0.65 [0.57, 0.73]	76.0	0.32	62.9	69.3
WHtR	0.66 [0.59, 0.74]	0.45	0.27	84.3	42.5
WHR	0.65 [0.57, 0.72]	0.81	0.30	81.4	49.0
Women					
BMI	0.59 [0.50, 0.67]^b^	24.72	0.25	58.5	67.0
WC	0.61 [0.53, 0.69]	75.0	0.29	69.8	58.7
WHtR	0.60 [0.52, 0.68]	0.52	0.18	62.3	55.8
WHR	0.67 [0.60, 0.74]	0.82	0.37	79.2	57.3

^a^The established obesity cut-off points are 30 kg/m^2^ for BMI, 90 cm (men) and 80 cm (women) for WC, 0.5 for WHtR, 0.9 (men) and 0.85 (women) for WHR

^b^AUCs for all obesity indices except BMI were significantly different from 0.50

^c^AUC for BMI of men was significantly lower than WC and WHtR (*p* < 0.01) and WHR (*p* < 0.05)

Table 3 shows the odds ratios of hypertension by obesity based on various obesity indices for men and women. Obese adults with WHtR ≥0.5 had about two times increased odds of having hypertension compared to non-obese adults. The association remained almost constant and significant in each model for both sexes. As for WC and WHR, significant adjusted odds ratios were observed in women only (models 2, 3, and 4). Meanwhile, none of the odds ratio (OR) for BMI was significant after controlling for the covariates.

**Table 3:** Association between obesity status and hypertension

Variables	Odds ratio [95% CI]			
	Model 1^a^	Model 2^b^	Model 3^c^	Model 4^d^
Men				
BMI ≥30 kg/m^2^	5.81 [1.10, 30.71]*	4.13 [0.76, 22.62]	3.93 [0.71, 21.65]	5.29 [0.94, 29.73]
WC ≥90 cm	2.96 [1.21, 7.27]*	2.79 [1.52, 5.14]**	2.45 [0.97, 6.14]	2.66 [1.05, 6.73]*
WHtR ≥0.5	2.46 [1.36, 4.46]**	2.17 [1.18, 3.98]*	2.11 [1.14, 3.90]*	2.15 [1.16, 4.01]*
WHR ≥0.9	2.18 [1.07, 7.23]*	1.59 [0.76, 3.36]	1.53 [0.72, 3.27]	1.59 [0.74, 3.42]
Women				
BMI ≥30 kg/m^2^	0.88 [0.34, 2.27]	1.35 [0.50, 3.64]	1.28 [0.47, 3.50]	0.99 [0.35, 2.82]
WC ≥80 cm	2.12 [1.15, 3.93]*	1.06 [1.03, 4.07]***	2.30 [1.20, 4.41]*	2.09 [1.07, 4.06]**
WHtR ≥0.5	2.05 [1.10, 3.80]*	2.13 [1.11, 4.07]*	2.09 [1.09, 4.02]*	1.97 [1.02, 3.82]*
WHR ≥0.85	2.62 [1.42, 4.85]**	1.06 [1.03, 1.08]***	1.99 [1.04, 3.80]*	1.94 [1.01, 3.73]*

^a^Model 1: unadjusted

^b^Model 2: adjusted for age

^c^Model 3: adjusted for age, smoking habit, and alcohol consumption

^d^Model 4: adjusted for age, smoking habit, alcohol consumption, and ethnicity (sub-tribes)

*p < 0.05, **p < 0.01, ***p < 0.001

## Discussion

In this population of Orang Asli adults, all obesity indices except BMI were able to predict hypertension, indicating that the complication of obesity is associated with the distribution of body fat rather than total body fatness. It would seem that WC, WHtR, and WHR were similarly useful as predictors of hypertension in men and women based on estimated AUC and unadjusted OR values. Nonetheless, when the effects of covariates were accounted, WHtR appeared to be the best parameter to predict the presence of hypertension. Previous studies showed that WHtR had the highest AUC and was most predictive of hypertension or metabolic risks, irrespective of sex and place of residence [38–41].

The measures of waist and height play a significant role in identifying the risk of obesity complications. WC is a reliable indicator of total abdominal fat with individuals having excessive abdominal fat that are at greater risk of cardiovascular disease [42]. Height could influence the distribution of body fat in that short people are more likely to deposit fat centrally. Several studies reported an inverse association between height and risk of cardiovascular disease [43, 44]. Stunting or short stature is prevalent in Orang Asli population, regardless of sex and age. More than half of adults in the present study were with short stature. As short stature is prevalent in Orang Asli, height should be accounted, in addition to waist circumference, for assessing the likelihood of at-risk individuals. In the present study, short stature could explain the better performance of WHtR as compared to waist circumference alone in predicting the presence of hypertension.

A study among Singaporean adults showed that both WC and WHtR as having equal power in predicting hypertension, irrespective of age, sex, and ethnicity [45]. The variation observed in our study as compared to the study may be due in part to the high prevalence of stunting in the Orang Asli population. The mean height of Singaporean men (170 ± 7 cm) and women (158 ± 6 cm) exceeded the cut-off value of short stature (men ≤160 cm; women ≤150 cm) [46], reflecting that stunting was not prevalent in that population. Hence, the performance of WC and WHtR in predicting hypertension was similar.

It should be noted that the proposed optimal WHtR cut-off point for hypertension in this study was 0.45 for men and 0.52 for women, approximating the established WHtR obesity cut-off point of 0.5. The smaller gap between the proposed and established obesity cut-off points for WHtR may further justify that it is better than other obesity indices in predicting the presence of hypertension in this population. Other studies reported similar finding in that WHtR could predict hypertension and/or other obesity complications at WHtR cut-offs ranged from 0.4 to 0.6 in non-indigenous and indigenous populations [40, 45, 47].

This study demonstrated that the predictive power of WHR on hypertension was relatively superior for women, but not for men. Similarly, the association between WHR and hypertension was reported to be more profound in Argentina women than men [48]. The variation in the predictive ability of WHR between men and women may relate to the difference in the impact of hormone on body fat distribution. Hormones drive the deposition of fat in the lower body part (pelvis, buttocks, and thighs) of women. A larger hip circumference was associated with a reduction of risk for multiple metabolic abnormalities in women only [49]. Therefore, WHR was more closely associated with hypertension in women than men.

We showed that BMI was a poor predictor of hypertension. Among indigenous peoples in Australia, obesity indices that reflect abdominal obesity performed better than BMI in predicting hypertension in both men and women [47]. A similar result was also observed in a population study of middle-aged Korean adults [41]. The poor performance of BMI to predict hypertension could be due to its inability to distinguish between muscle mass and adipose tissue, with the latter being more relevant to metabolic risk. A study in Vietnamese adults reported that percent body fat was linearly related to BMI among adults with BMI <27, but the relationship was leveled off for individuals with BMI >27 [50]. A weak association between percent body fat and BMI was found to be more prominent among females and Asians. At the same BMI threshold, women and Asians had relatively higher percent body fat than other groups [51, 52].

Several limitations to the present study should be highlighted. First, this study attempted to assess the relationship between indices of body adiposity and hypertension. As these indices may have limitations as measures of body adiposity, their associations with hypertension could be under or over-estimated. Second, the “predictive ability” in this study refers to the ability to detect the presence of hypertension but not the ability to predict future development of hypertension. Third, a BMI cut-off point of 30 kg/m^2^ defines obesity. In Asian population, a lower BMI cut-off might be more appropriate to assess the association between obesity and cardiometabolic risk factors, such as hypertension. For instance, Asians might have a high risk of developing cardiovascular diseases at a BMI of 27.5 kg/m^2^ [53]. We excluded subjects with medical diagnosis of chronic diseases (cancer, cardiovascular disease, diabetes, and renal diseases) that could confound the association between obesity and presence of hypertension. However, there could be undiagnosed cases of such diseases with presence of secondary hypertension as well as unreported diagnosed cases that were included in the study. While the study addressed several biological (age and sub-tribes) and lifestyle (smoking and alcohol consumption) covariates in the analyses, other potential covariates are not included. The cross-sectional design of this study and the inclusion of only three sub-ethnic groups in a specific locality could also limit the causal inference of obesity—hypertension relationship as well as the generalizability of study findings, respectively.

## Conclusions

Hypertension is a growing but often a hidden health problem in Orang Asli population. A majority of hypertensive Orang Asli adults are less likely to be diagnosed, mainly due to lack of regular access to primary health care. As WHtR can easily be measured and determined, it can be used as a screening tool by health professionals to identify Orang Asli at risk of hypertension and refer them for further diagnostic evaluation and subsequent medical treatment.

## Abbreviations

AUC: Area under the ROC curve
BMI: Body mass index
DBP: Diastolic blood pressure
NHMS: National Health and Morbidity Survey
OR: Odds ratio
ROC: Receiver operating characteristic
SBP: Systolic blood pressure
WC: Waist circumference
WHO: World Health Organization
WHR: Waist-hip ratio
WHtR: Waist-height ratio
